# DNA Nanostructures in the Fight Against Infectious Diseases

**DOI:** 10.1002/anbr.202000049

**Published:** 2021-01-06

**Authors:** David M. Smith, Adrian Keller

**Affiliations:** ^1^ DNA Nanodevices Unit Department Diagnostics Fraunhofer Institute for Cell Therapy and Immunology IZI 04103 Leipzig Germany; ^2^ Peter Debye Institute for Soft Matter Physics Faculty of Physics and Earth Sciences University of Leipzig 04103 Leipzig Germany; ^3^ Institute of Clinical Immunology University of Leipzig Medical School 04103 Leipzig Germany; ^4^ Dhirubhai Ambani Institute of Information and Communication Technology Gandhinagar 382 007 India; ^5^ Technical and Macromolecular Chemistry Paderborn University Warburger Str. 100 33098 Paderborn Germany

**Keywords:** detection, DNA nanotechnologies, infectious diseases, inhibitions, nanomedicine, pathogens

## Abstract

Throughout history, humanity has been threatened by countless epidemic and pandemic outbreaks of infectious diseases, from the Justinianic Plague to the Spanish flu to COVID‐19. While numerous antimicrobial and antiviral drugs have been developed over the last 200 years to face these threats, the globalized and highly connected world of the 21st century demands for an ever‐increasing efficiency in the detection and treatment of infectious diseases. Consequently, the rapidly evolving field of nanomedicine has taken up the challenge and developed a plethora of strategies to fight infectious diseases with the help of various nanomaterials such as noble metal nanoparticles, liposomes, nanogels, and virus capsids. DNA nanotechnology represents a comparatively recent addition to the nanomedicine arsenal, which, over the past decade, has made great progress in the area of cancer diagnostics and therapy. However, the past few years have seen also an increasing number of DNA nanotechnology‐related studies that particularly focus on the detection and inhibition of microbial and viral pathogens. Herein, a brief overview of this rather young research field is provided, successful concepts as well as potential challenges are identified, and promising directions for future research are highlighted.

## Graphical abstract

Infections arising from pathogenic organisms such as viruses and bacteria represent a dire threat to modern society due to their impact on human health as well as the potential for economic and societal disruption in the case of global pandemics. Approaches to detect and treat infectious diseases based on DNA nanostructures offer many promising, although still widely unexplored, possibilities.

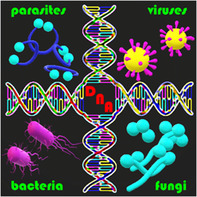

## 1 Introduction

The ongoing COVID‐19 pandemic has once more led to the realization that humanity is dangerously ill prepared for fighting the threats associated with epidemic outbreaks of infectious diseases. COVID‐19 is only the latest in a long list of viral diseases, including SARS,^[^ ^1^ ^]^ MERS,^[^ ^2^ ^]^ Zika,^[^ ^3^ ^]^ Ebola,^[^ ^4^ ^]^ and Nipah,^[^ ^5^ ^]^ that emerged as severe threats to public health in the past two decades and that continue to threaten human lives. However, also diseases that have plagued humanity for centuries still present a severe burden. For instance, the 20th century has seen three influenza pandemics with death tolls in the millions^[^ ^6^ ^]^ and the seasonal flu still kills on average an estimated 389 000 people each year.^[^ ^7^ ^]^ Furthermore, due to the global rise of antibiotic resistance in the past few decades, bacterial infections have once again become a severe threat to human health.^[^ ^8^ ^]^ In 2015, about 670 000 patients in the EU alone suffered from infections with antibiotic‐resistant strains of eight bacterial pathogens, resulting in 33 000 deaths.^[^ ^9^ ^]^ The situation in the US is similar, with an estimated 23 000 deaths each year as a direct result of more than 2 million multidrug‐resistant infections.^[^ ^10^ ^]^ In low‐ and middle‐income countries with limited ability to pay for second‐line drugs, antibiotic resistance results in even higher mortality rates. In 2010, about 38 000 deaths in Thailand alone were attributed to infections with only five antibiotic‐resistant bacteria.^[^ ^11^ ^]^ In India, it is estimated that about 60 000 neonatal deaths each year result from antibiotic‐resistant bacterial infections.^[^ ^8^ ^]^ More recently, the worldwide emergence of antifungal resistance has raised grave concerns as well,^[^ ^12^ ^]^ as invasive fungal infections are responsible for at least 2 million life‐threatening infections each year with mortality rates often exceeding 50%.^[^ ^13^ ^]^ However, the infectious disease responsible for the largest number of fatalities worldwide is still tuberculosis, which caused the deaths of 1.5 million people in 2018.^[^ ^14^ ^]^

These examples make it clear that the fight against infectious diseases has only just begun. In fact, only one infectious disease so far deserves the distinction “eradicated.”^[^ ^15^ ^]^ Forty years ago, in May 1980, WHO officially declared the eradication of smallpox,^[^ ^16^ ^]^ 13 years after the start of its intensified eradication program and 180 years after the discovery of the first smallpox vaccine.^[^ ^17^ ^]^ While this was a tremendous feat, in today's globalized world, where a previously unknown disease such as COVID‐19 can spread around the globe within weeks, we need to respond much more rapidly to epidemic outbreaks of old and newly emerging infectious diseases. To this end, novel diagnostic tools, therapeutic approaches, and preventive measures are urgently needed.

The advent and rise of biomedical nanotechnology has sparked new hope for the treatment of severe diseases for which traditional therapies have ended in a standstill.^[^ ^18^, ^19^ ^]^ As of now, about 30 therapeutic and diagnostic nanoparticles have received clinical approval by the U.S. Food and Drug Administration or European Medicines Agency, and more than 60 are currently in clinical trials.^[^ ^20^ ^]^ However, the vast majority of these nanoparticle‐based formulations are aimed at cancer therapy, whereas only a few specifically target infectious diseases. Even though the potential of liposomal micro‐ and nanoparticles to serve as drug carriers in the treatment of infectious diseases was recognized rather early,^[^ ^21^ ^]^ the most drastic developments have been made in the field of pathogen detection and ex vivo diagnostics.^[^ ^22^ ^]^ This has spawned a multitude of nanotechnology‐based diagnostic tests and assays for numerous severe and often fatal diseases such as hepatitis C,^[^ ^23^ ^]^ dengue fever,^[^ ^24^ ^]^ and tuberculosis.^[^ ^25^ ^]^ Nevertheless, the past two decades have seen progress also in the application of nanoparticles in infectious disease treatment^[^ ^26^ ^]^ and vaccine development.^[^ ^27^ ^]^ Consequently, a few nanoparticle‐based formulations against infectious diseases such hepatitis B, pneumonia, and chikungunya have entered clinical trials in the past few years,^[^ ^20^ ^]^ not to mention the numerous COVID‐19 vaccine candidates.^[^ ^28^ ^]^

When it comes to drug and gene delivery applications, the field is still dominated by lipid nanoparticles and in particular liposomes,^[^ ^29^ ^]^ whereas a plethora of diagnostic assays and biosensors rely on the optical properties of noble metal nanoparticles.^[^ ^30^ ^]^ DNA nanotechnology, i.e., the repurposing of DNA as a construction material, on the other hand represents one of the newer additions to the nanomedicine arsenal. A large variety of biomedical DNA nanostructures have been reported in literature, ranging from small aptamers^[^ ^31^ ^]^ and triple helices^[^ ^32^ ^]^ to medium‐sized DNA tetrahedra^[^ ^33^ ^]^ to large tile‐based and DNA origami nanostructures.^[^ ^34^, ^35^ ^]^ Such nanostructures can be composed of short synthetic oligonucleotides,^[^ ^36^ ^]^ biochemically or genetically engineered long DNA single strands,^[^ ^37^ ^]^ or a combination of both.^[^ ^38^ ^]^ The overall geometry and shape of the assembled DNA nanostructures are determined by the nucleotide sequences of their single‐stranded components, which are designed to facilitate multiple hybridization events. Numerous software solutions are available to aid in the design of the desired DNA nanoshapes and simulate their mechanical properties and stability.^[^ ^39^ ^]^ From an application‐oriented standpoint, the most intriguing feature of DNA nanostructures is the possibility to chemically modify selected DNA single strands and thereby arrange functional species such as functional nucleic acids,^[^ ^40^ ^]^ fluorophores,^[^ ^41^ ^]^ proteins,^[^ ^42^ ^]^ and nanoparticles,^[^ ^43^ ^]^ with a precision down to the subnanometer level.^[^ ^44^, ^45^ ^]^ This enables the introduction of sensor, actuator, and transducer elements and thus the creation of DNA nanodevices that can sense their surrounding environment, interact with it, and respond to environmental changes. In the past decade, this versatility of DNA nanostructures has been exploited in numerous application fields, including nanoelectronics,^[^ ^46^ ^]^ (bio)sensing,^[^ ^47^ ^]^ and biomedicine.^[^ ^34^ ^]^

Even though DNA nanostructures are intrinsically less stable than more established nanoparticles,^[^ ^48^ ^]^ the exceptionally high degree of structural and functional control they provide makes them promising candidates for applications in targeted drug delivery and disease biomarker detection. DNA nanostructure‐based drug delivery vehicles and sensing devices thus have been investigated extensively in vitro and in vivo and impressive progress has been made.^[^ ^34^ ^]^ The vast majority of these studies were traditionally aimed at applications in cancer therapy and diagnostics,^[^ ^33^, ^49^ ^]^ whereas the field of infectious diseases has largely been neglected.^[^ ^34^ ^]^ Recently, however, this situation began to change with an increasing number of DNA nanotechnology‐related papers addressing specific aspects in infectious disease treatment and pathogen detection. This review thus aims at summarizing the recent advancements made in this young and evolving field, highlighting particularly promising developments, and identifying new research directions. Note that we will primarily focus on nanoparticles with a clearly defined 3D shape that are based on DNA as the scaffolding material. Detection and delivery concepts utilizing simple duplex DNA^[^ ^50^ ^]^ or aptamer‐conjugated non‐DNA nanoparticles^[^ ^51^ ^]^ are not considered in the remainder of the article.

## 2 DNA Nanostructures for Pathogen Detection

Numerous biosensing and diagnostic strategies based on DNA nanostructures have been reported in literature.^[^ ^52^ ^]^ The vast majority of these strategies have focused on tumor diagnostics^[^ ^53^ ^]^ and microRNA (miRNA) detection.^[^ ^54^ ^]^ However, the past few years have seen several adaptions of such DNA nanostructure‐based sensing concepts for the detection of various pathogens. This development is mostly motivated by the increasing demand for simple point‐of‐care diagnostic devices that do not rely on expensive external equipment.^[^ ^55^ ^]^ Several recent epidemic outbreaks of severe infectious diseases in remote areas contributed to this increased demand, such as the 2014 Ebola outbreak in Western Africa,^[^ ^56^ ^]^ the 2015 Zika outbreak in Latin America,^[^ ^57^ ^]^ and the 2017 plague outbreak in Madagascar.^[^ ^58^ ^]^ In the face of the COVID‐19 pandemic, the search for novel point‐of‐care diagnostic concepts has gained additional momentum.^[^ ^59^ ^]^ In this section, we will thus discuss some promising DNA nanostructure‐based concepts for the detection of various infection‐related biomarkers and complete pathogens. The application of DNA nanostructures in the detection of microbial toxins as well as food and water contaminants is not considered in this regard. For this, the reader is referred to some recent reviews that focus specifically on these topics.^[^ ^60^ ^]^

### 2.1 Nucleic Acid Biomarkers

Since they are entirely based on DNA, most pathogen‐sensing concepts use DNA nanostructures for the selective binding of pathogen‐specific nucleic acid biomarkers. However, most of these works have used synthetic target DNA but no clinical or patient samples. The latter will present a number of additional challenges, in particular with regard to point‐of‐care applications, and usually require comparatively complex, expensive, and time‐consuming polymerase chain reaction (PCR) amplification.^[^ ^61^ ^]^ An overview of the different nucleic acid‐sensing concepts discussed in this section is shown in **Table** **1**.

**Table 1:** Overview and comparison of the different nucleic acid‐sensing concepts

Method	Target pathogen	LOD	Comment	Reference
Dendrimer‐based fluorescent barcodes	*Bacillus anthracis*, *Francisella tularensis*, Ebola virus, SARS coronavirus	620 amol	Compatible with various detection techniques (microbead‐based fluorescence microscopy, blotting, flow cytometry)	[62]
Dendrimer formation by HCR	HIV, *Chlamydia trachomatis*	50 fmol	Detection by gel electrophoresis; optical detection may be possible	[64]
Fluorescent beacons attached to plasmonic nanoantennae	Zika virus	≤1 nM	Detection in target‐enriched human serum	[65]
Fluorescence‐quenching DNA tweezer	IAV	100–200 nM	Multiplexed detection of DNA sequences from different IAVs in a single mixture	[68]
CD	HCV	100 pM	Detection in diluted serum	[70]
AFM detection of bound targets	HPV	≤100 nM	PCR‐amplified genes obtained from clinical samples	[72]
AFM of target‐bound shape barcodes	HBV	10 pM	Genotyping of clinical samples	[73]
Electrochemical detection	IAV	97 fM	Asymmetric PCR products from clinical throat‐swab samples; detection after only one PCR cycle demonstrated	[74]

**Figure 1:**
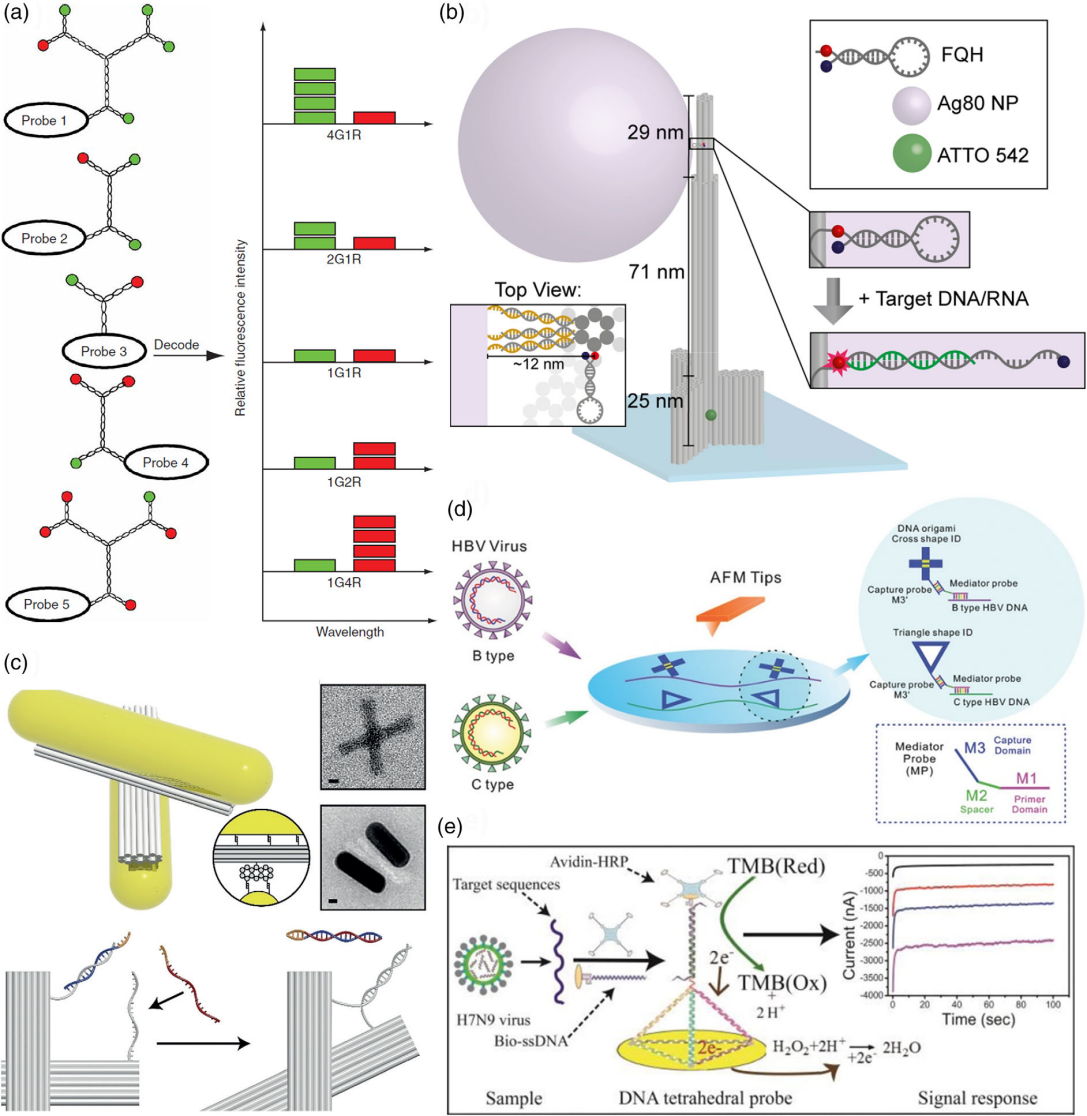
Strategies for the DNA nanostructure‐based detection of nucleic acid biomarkers. a) Dendrimeric fluorescent DNA barcodes. Reproduced with permission.^[^ ^62^ ^]^ Copyright 2005, Springer Nature. b) Plasmonic DNA origami nanoantenna for the detection of Zika nucleic acids using FQHs. Reproduced with permission.^[^ ^65^ ^]^ Copyright 2017, American Chemical Society. Further permission related to the material excerpted should be directed to the American Chemical Society. c) A switchable DNA origami device carrying AuNRs for the detection of HCV‐specific RNA by CD spectroscopy. Reproduced with permission.^[^ ^70^ ^]^ Copyright 2018, John Wiley and Sons. d) Topological DNA origami barcodes for the genotyping of HBV. Reproduced with permission.^[^ ^73^ ^]^ Copyright 2018, John Wiley and Sons. e) DNA tetrahedron on a gold electrode for the electrochemical detection of IAV genes using a HRP redox probe. Reproduced with permission.^[^ ^74^ ^]^ Copyright 2015, American Chemical Society.

As an early example for the application of DNA nanostructures in pathogen detection, Li et al. demonstrated the multiplexed detection of DNA sequences specific for different pathogens using fluorescent DNA nanostructure barcodes.^[^ ^62^ ^]^ These barcodes were based on DNA three‐arm junctions assembled by sticky‐end hybridization and subsequent ligation into well‐defined dendrimer‐like DNA nanostructures. Using a selection of fluorescently labelled three‐arm junctions, different DNA barcodes were synthesized that carried different numbers of the two fluorophores and could thus be distinguished by the relative intensities of the two fluorescence wavelengths (see **Figure** **1**a). Each barcode was further designed to carry a different single‐stranded capture probe to hybridize to a DNA sequence specific for one of four different pathogens. In this way, the authors were able to specifically detect DNA sequences of the pathogens *Bacillus anthracis*, *Francisella tularensis*, Ebola virus, and SARS coronavirus in parallel from a complex DNA mixture. Target specificity was verified against controls of DNA single strands with irrelevant sequences and plasmid DNA. The authors further demonstrated the compatibility of these DNA nanostructure barcodes with a number of detection techniques such as microbead‐based fluorescence microscopy, blotting, and flow cytometry. For the flow cytometry assay, a limit of detection (LOD) of 620 amol was determined.

While Li et al. used DNA dendrimers of defined sizes for the assembly of fluorescent barcodes,^[^ ^62^ ^]^ Chandran et al. rather used dendrimer assembly by a target DNA‐triggered hybridization chain reaction (HCR)^[^ ^63^ ^]^ to amplify binding events.^[^ ^64^ ^]^ In this way, the hybridization of a short, target DNA sequence resulted in the formation of a high‐molecular‐weight dendrimer that could be detected by gel electrophoresis. The authors demonstrated the detection of DNA sequences from human immunodeficiency virus (HIV) and *Chlamydia trachomatis* and determined a LOD of 50 fmol. While the assay showed specificity for the target strand over an arbitrary DNA sequence with no sequence similarity to the target, it could not distinguish between the fully matched target sequence and mutated sequences carrying 1–3 mismatches.

A different approach for the amplification of binding events was demonstrated by Ochmann et al.^[^ ^65^ ^]^ They used fluorescence‐quenching hairpins (FQHs), so‐called molecular beacons,^[^ ^66^ ^]^ which are opened upon target binding, resulting in a detectable fluorescence signal. They combined these hairpins with silver nanoparticle (AgNP)‐modified DNA origami nanostructures, which act as plasmonic nanoantennae and can thereby enhance the fluorescence of the used dye by several orders of magnitude (see Figure 1b).^[^ ^67^ ^]^ Using this approach, the authors demonstrated the detection of Zika virus‐specific DNA and RNA sequences with concentrations of 1 nM. This sensing approach showed high target sensitivity with two mismatches in the target sequence resulting in signal reduction by more than 50%. DNA detection was achieved also in target‐enriched human serum and multiplexing was demonstrated as well.

Fluorescence quenching‐based target detection was also used by Lertanantawong et al.^[^ ^68^ ^]^ The authors designed a tweezer‐like DNA nanostructure with a fluorophore–quencher pair at the distal ends of the two arms. Target DNA binding initiated a stand displacement reaction (SDR),^[^ ^69^ ^]^ which led to a closed conformation of the tweezer and thereby the quenching of the fluorescence signal. Using three tweezers carrying three different fluorophores, the authors demonstrated the multiplexed detection of DNA sequences specific for three different influenza A viruses (IAVs) from a single mixture. Target specificity was verified against non‐target IAV genes. The LOD was determined to be 100–200 nM.

As an alternative optical detection method, Funck et al. used circular dichroism (CD) spectroscopy for the detection of a hepatitis C virus (HCV)‐specific RNA sequence.^[^ ^70^ ^]^ They used a cross‐shaped DNA origami nanostructure based on two rigid arms connected with a flexible pivot point (see Figure 1c). Two plasmonic gold nanorods (AuNRs) were attached to the arms, which, in the absence of the target RNA, had a crossed and almost achiral conformation. Addition of target RNA then initiated an SDR that led to a change of this conformation. In the switched state, the two AuNRs were in a right‐handed chiral conformation. Due to the plasmonic coupling of the two AuNRs, this switched state led to a pronounced CD signal. Target specificity was demonstrated against a noncomplementary random RNA sequence. A LOD of 100 pM was determined in buffer, which is comparable with other RNA detection methods but still 10–50 times higher than the average viral load in patient blood. Target RNA detection was demonstrated also in diluted serum.

Atomic force microscopy (AFM) is an established technique for the characterization of DNA nanostructures and can also be used for the detection and site‐specific visualization of target molecules bound to capture probes immobilized on the DNA nanostructure surface.^[^ ^44^, ^71^ ^]^ Li et al. used this approach to detect PCR‐amplified human papillomavirus (HPV) genes obtained from clinical samples at 100 nM concentration.^[^ ^72^ ^]^ The target sequence was hybridized to two capture sequences protruding from the surfaces of DNA origami nanostructures and AFM was used to detect the resulting duplex DNA. In a different approach, Liu et al. used AFM to determine the genotypes of hepatitis B viruses (HBV).^[^ ^73^ ^]^ The authors obtained a 1 kb single‐stranded DNA fragment of the HBV genome containing the S gene region from human serum samples by asymmetric PCR and enzymatic digestion. They then hybridized two specifically designed mediator probes MP1 and MP2 (see Figure 1d) to two regions of the fragment that carry a number of mutations specific to genotypes B and C, resulting in short double‐stranded (ds) regions with single‐stranded overhangs. Afterward, the hybridized mediator probe sequences M1 were elongated in a polymerase reaction, which produced a dsDNA target template with two single‐stranded overhangs (M3). Two different DNA origami shapes were then hybridized to the M3 overhangs via complementary sticky ends with a cross and a triangular shape corresponding to the B and C genotype, respectively (see Figure 1d). The identity of the DNA origami labels attached to the target templates was determined by AFM. Using clinical samples, a LOD of 10 pM was determined for this assay, which was superior to other established assays such as Abbott real‐time PCR or TaqMan assays. Furthermore, the specificity of the assay was assessed in a single blind test against 11 unknown serum samples and verified by capillary sequencing of the PCR products.

An electrochemical sensing approach toward pathogen DNA detection was presented by Dong et al.^[^ ^74^ ^]^ The authors immobilized thiol‐modified DNA tetrahedra at gold electrodes, each of which displayed a single‐stranded probe sequence for the immobilization of an IAV gene. After target DNA binding, a biotinylated oligonucleotide was hybridized to a single‐stranded overhang of the target DNA and used to immobilize an avidin‐conjugated HRP enzyme (see Figure 1e). Enzyme activity at the gold electrode surface was then monitored by amperometry. This sensor was highly specific for the target sequence and could distinguish the target sequence not only from asymmetric PCR products of different IAVs but even from mutated sequences with single mismatches. It also showed better performance than a similar sensor based only on thiolated oligonucleotides as capture probes. Most importantly, the authors tested their sensor against clinical IAV‐containing throat‐swab samples and successfully detected asymmetric PCR products with a LOD of 97 fM. However, the authors also demonstrated the detection of asymmetric PCR products after only one PCR cycle. This represents an important step toward the PCR‐free detection of viral nucleic acids in clinical samples.

### 2.2 Protein Biomarkers

**Figure 2:**
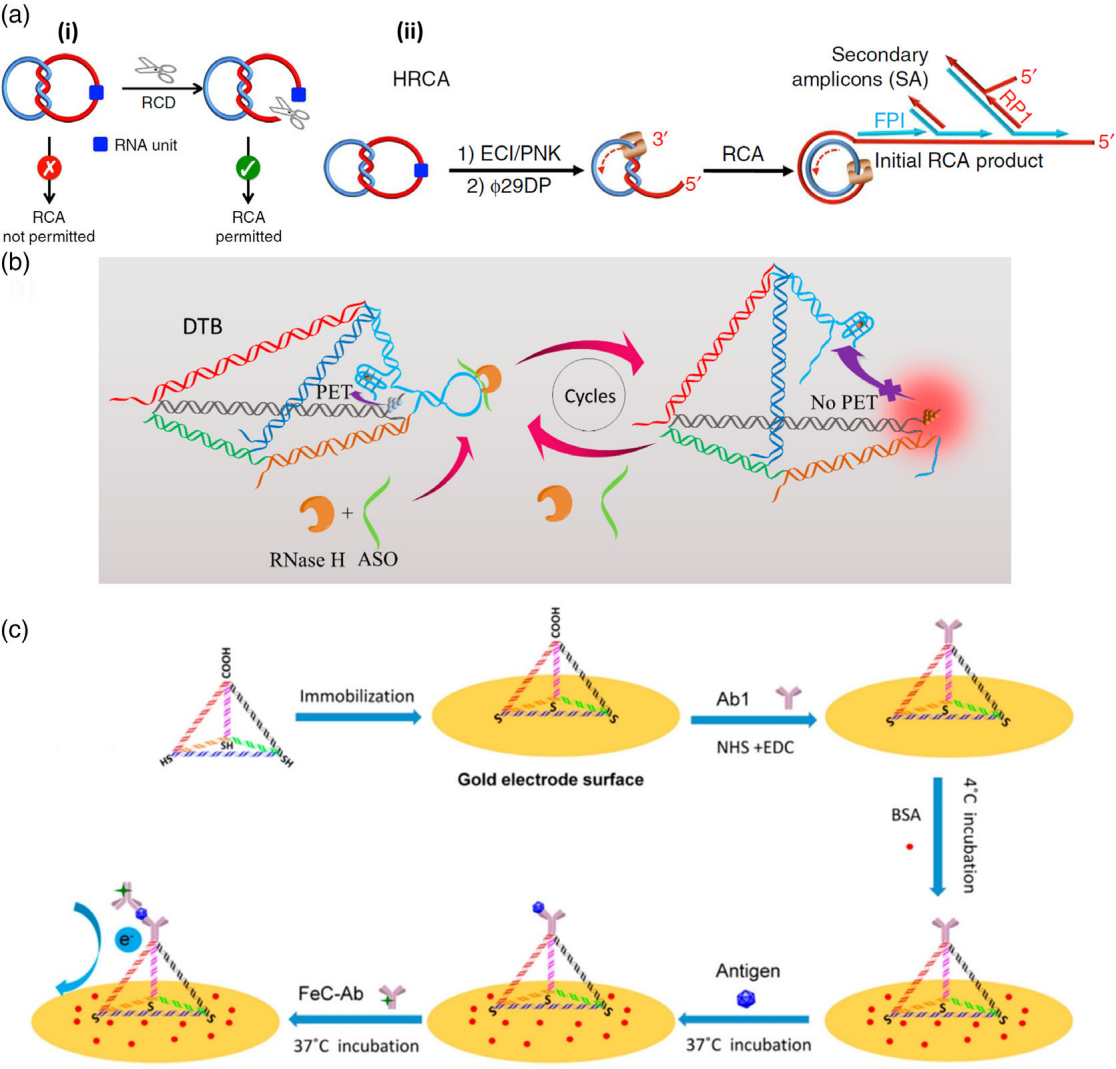
Strategies for the DNA nanostructure‐based detection of protein biomarkers. a) An RCA‐resistant DNA catenane can be activated by a DNAzyme that cleaves an RNA recognition site (blue) incorporated in one of the rings (panel i). The combination of HRCA with a DNAzyme that is activated only in the presence of a certain protein biomarker (panel ii) enables *E. coli* detection. Reproduced under the terms of the Creative Commons Attribution 4.0 International License.^[^ ^75^ ^]^ Copyright 2016, The Authors. b) A DNA tetrahedron carrying a fluorophore quencher close to a hairpin loop modified with an RNA sequence. Hybridization of an ASO enables the cleavage of the loop by HIV RNase H, which restores fluorescence. Reproduced with permission.^[^ ^78^ ^]^ Copyright 2019, American Chemical Society. c) An antibody‐modified DNA tetrahedron on a gold electrode for the electrochemical detection of *S. pneumoniae*‐specific PspA protein using a FeC‐labeled antibody as a redox probe. Reproduced with permission.^[^ ^81^ ^]^ Copyright 2017, American Chemical Society.

The detection of protein biomarkers by DNA nanostructures is more complex as it requires the incorporation of a protein‐specific recognition element. Recognition elements can be nucleic acids, proteins, or small molecules. Liu et al. presented an approach that utilized a short RNA segment in a DNA catenane composed of two mechanically interlocked single‐stranded DNA circles with a linking duplex.^[^ ^75^ ^]^ In its original form, this DNA catenane was resistant toward rolling‐circle amplification (RCA).^[^ ^76^ ^]^ In the presence of an active RNA‐cleaving DNAzyme, i.e., a single‐stranded catalytic DNA sequence that cleaves a specific RNA substrate,^[^ ^77^ ^]^ however, the RNA segment was cleaved, which linearized one of the two rings and thereby enabled RCA of the still intact ring (see panel (i) of **Figure** **2**a). Using a DNAzyme that is activated only in the presence of an *Escherichia coli*‐produced protein, the authors were able to detect *E. coli* at a concentration of ten cells per ml in whole blood. This was achieved by fluorescence detection of DNA products from a hyperbranched RCA reaction (HRCA, see panel (ii) of Figure 2a). By comparison, an enzyme‐linked immunosorbent assay (ELISA) had a 100 times higher LOD under equivalent conditions. Furthermore, target specificity of the assay was verified against four other Gram‐negative and three Gram‐positive bacteria as well as the total small RNAs extracted from breast cancer cell line MCF‐7.

Zhang et al. used an RNA recognition site in a DNA tetrahedron (see Figure 2b).^[^ ^78^ ^]^ Here, the RNA sequence was included in the loop region of a hairpin structure formed at one of the tetrahedron's edges. This hairpin brought a fluorescent silver nanocluster (AgNC) into the close vicinity of a fluorescence‐quenching G quadruplex. Because of the strain exerted by the hairpin on the other edges of the tetrahedron, enzymatic cleavage of the RNA site in the hairpin loop resulted in the dissociation of the hairpin stem and thus a larger separation of the AgNC–quadruplex pair, which was detectable by an increase in AgNC fluorescence. As the tetrahedron could cross cell membranes, this assay enabled the intracellular detection of the argonaute2 (Ago2) protein, which plays a role in RNA interference (RNAi). However, using a modified version of their assay (see Figure 2b), the authors also demonstrated the detection of ribonuclease (RNase) H, which is involved in the reverse transcriptase pathway of HIV type‐1 (HIV‐1). A LOD of 3.41 U RNase H per ml was determined, which places this sensing approach among the more sensitive RNase H assays reported in literature. Target specificity for RNase H was verified against a number of other proteins, i.e., Ago1, Ago2, Ago3, and EcoR1. Furthermore, quantitative RNase H detection was demonstrated in several cell lysates.

Specific detection of protein biomarkers can also be achieved using aptamers, i.e., DNA or RNA sequences that specifically bind a target molecule under certain environmental conditions.^[^ ^79^ ^]^ Godonoga et al. decorated a DNA origami nanostructure with DNA aptamers against the malaria‐specific protein biomarker *Pf*LDH.^[^ ^80^ ^]^ *Pf*LDH detection was achieved at a protein concentration of 500 nM by AFM and target specificity was verified against the human homolog hLDH. Target binding to the DNA origami nanostructures was also demonstrated in human blood plasma.

Instead of an aptamer, Wang et al. covalently attached a target‐specific antibody to one tip of a DNA tetrahedron that was immobilized via thiol modifications on the surface of a gold electrode.^[^ ^81^ ^]^ The antibody was then used to capture pneumococcal surface protein A (PspA) from *Streptococcus pneumoniae* lysate. Once bound, a second anti‐PspA antibody was attached to the protein. This particular second antibody was labelled with an electroactive ferrocene carboxylic acid (FeC) tag, so that its binding to the immobilized protein could be detected using square wave voltammetry. The LOD of this assay was determined as 0.093 CFU per mL equivalent of *S. pneumoniae* lysate, which was 1–2 orders of magnitude lower than the LOD of other PCR‐based assays. Target specificity was verified against *E. coli* lysate and bovine serum albumin (BSA). Furthermore, the authors also managed to detect PspA in swab samples obtained from the nasal cavity and mouth of a human subject, whereas the swab sample from the axilla tested negative for *S. pneumoniae* in accordance with literature. Remarkably, *S. pneumoniae* detection in swab samples was achieved with minimal sample processing and without any amplification or purification.

### 2.3 Whole Pathogens

**Figure 3:**
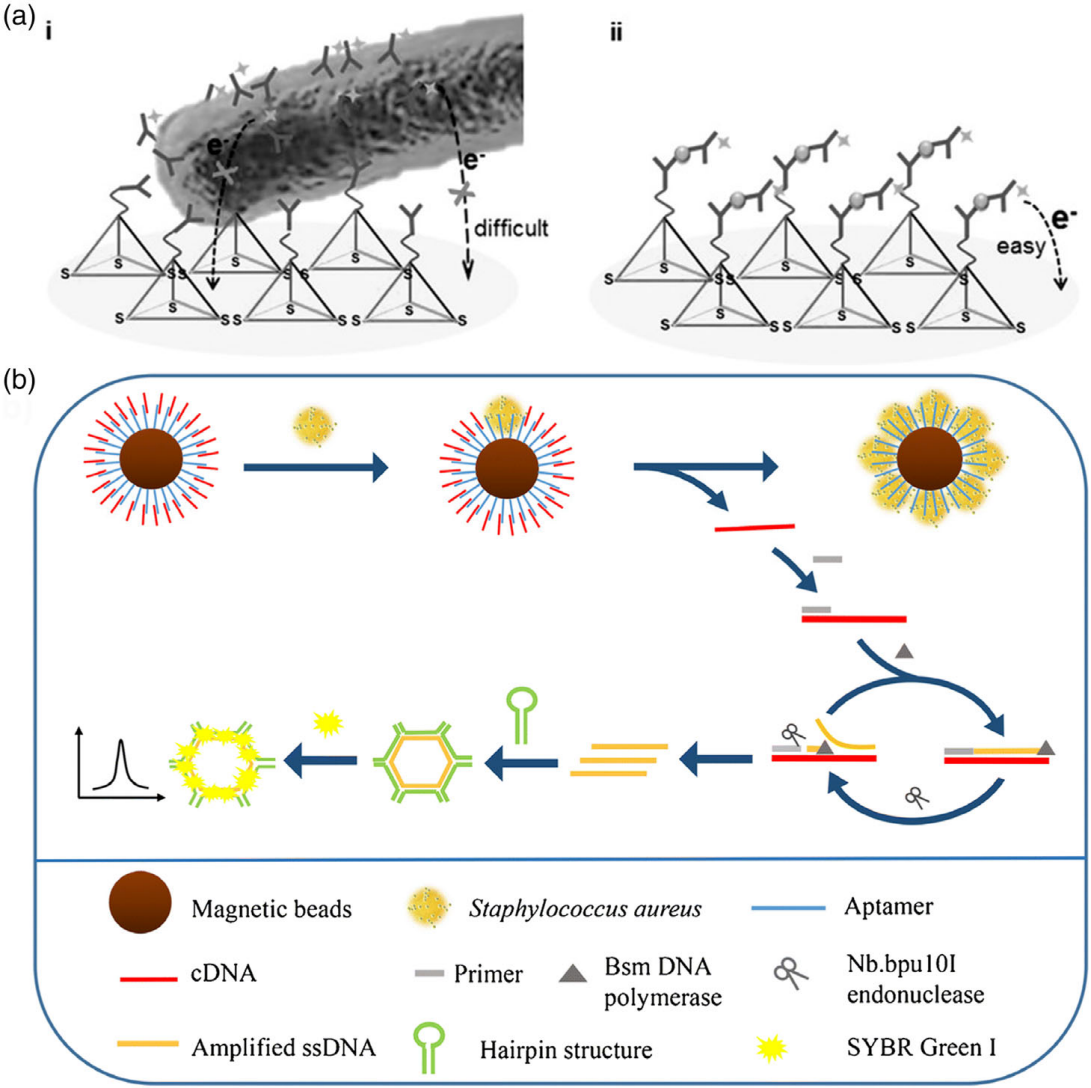
Strategies for the DNA nanostructure‐based detection of whole pathogens. a) Electrochemical detection of *E. coli* by antibody‐modified DNA tetrahedra on a gold electrode and FeC‐labeled antibodies as a redox probe. Due to the large size of the bacterium (panel i), charge transport between the probe antibodies and the electrode surface is hindered. This problem does not occur when detecting molecular biomarkers from cell lysate (panel ii). Reproduced with permission.^[^ ^82^ ^]^ Copyright 2015, John Wiley and Sons. b) Upon binding of *S. aureus*, a DNA template is released from aptamer‐modified magnetic beads and multiplied by the interplay of polymerase and endonuclease enzymes. The amplified DNA strands open hairpins and facilitate their assembly into DNA hexagons, which are loaded with an intercalating dye for fluorescence detection. Reproduced with permission.^[^ ^83^ ^]^ Copyright 2020, Springer Nature.

Only very few studies have attempted to detect whole (live) pathogens with the help of DNA nanostructures because adapting molecular sensing approaches to such large targets often faces significant challenges. For instance, Giovanni et al. used an electrochemistry‐based approach conceptually similar to the one reported by Wang et al. that was discussed earlier (see Figure 2c).^[^ ^82^ ^]^ In this particular implementation, however, the authors used antibodies specific against the lipopolysaccharide (LPS) found in the membrane of *E. coli* bacteria. Using the sandwich‐type assay, a LOD for *E. coli*‐derived LPS of 0.20 ng ml^−1^ was determined. The same assay proved capable also of detecting *E. coli* lysates with a LOD of 1.20 CFU ml^−1^, which is lower than that of other (amplification‐free) electrochemical detection methods. Specificity for all targets was verified also in the presence of 1% skim milk. Finally, the authors also demonstrated the detection of whole *E. coli* bacteria. However, while their sensor was able to detect even a single bacterium, the concentration‐dependent linear sensor response was much weaker than that for LPS and lysate detection. This was attributed to the large size of the bacteria, which can hamper the detection of redox currents (see **Figure** **3**a). Even though each bacterium can capture multiple FeC‐modified antibodies, it will also bind to several DNA tetrahedra at the electrode surface. Furthermore, its micron‐sized bulk represents a nonconductive barrier between the electrode surface and FeC label, which blocks electron transfer between them. Therefore, while this sensing approach may be useful for detecting low amounts of bacteria, lysate detection is more suitable for the quantification of bacteria concentrations.

Recently, Cai et al. presented a fluorescence‐based assay for the specific detection of *Staphylococcus aureus* that does not suffer from such limitations (see Figure 3b).^[^ ^83^ ^]^ The authors coated magnetic beads with an *S. aureus*‐specific aptamer that was partially hybridized to a complementary DNA (cDNA). Binding to the *S. aureus* target resulted in a conformational transition in the aptamer and the release of cDNA, upon which it could bind to a short primer sequence. The primer was then elongated by a DNA polymerase. The extended primer contained a restriction site that was recognized and cleaved by an endonuclease, providing a new priming site for polymerase. During the second extension of the primer, the cleaved fragment was dissociated from the template cDNA. The repetition of this polymerase–nuclease cycle generated a large number of copies of the fragment, which were subsequently assembled into a hexagonal‐shaped DNA nanostructure upon hybridization with six DNA hairpins. Using an intercalating fluorescent dye, a fluorescence signal proportional to the amount of generated DNA hexagons was obtained. At a LOD of 1.7 CFU ml^−1^, this assay was more sensitive than most other assays reported in literature. At the same time, it had a larger dynamic range than the other methods. Furthermore, the assay was highly specific for *S. aureus* over several other Gram‐positive and Gram‐negative bacteria and detection was also demonstrated in milk samples.

## 3 DNA Nanostructures for Pathogen Inhibition

The implementation of DNA‐ and other nucleotide‐based nanostructures in strategies for the passive or targeted delivery of therapeutic agents has been one of the most overt motivations behind the biologically driven development of the technology.^[^ ^35^, ^84^ ^]^ In comparison with the use of other types of nanoparticles made from biological or inorganic materials,^[^ ^18^, ^20^ ^]^ the use of nucleic acids holds multiple advantages. Beyond the inherent biocompatibility and potential for biodegradability after the therapeutic agent has been delivered to or acted upon its target, the ability to arrange exact numbers of active biomolecules on the underlying nucleic acid structure in precise arrangements is a unique feature unavailable in other nanoparticle systems. This allows active, therapeutic molecules to be combined with additional targeting or stimulatory moieties in a manner rationally designed to enhance the overall efficacy. Indeed, several preliminary studies on both cellular^[^ ^85^, ^86^, ^87^, ^88^ ^]^ and animal models^[^ ^86^, ^89^, ^90^ ^]^ have pointed toward the future promise of this approach. Nevertheless, relatively few studies have focused on battling pathogenic infections; rather, cancer has been the most popular target for DNA‐based particles to deliver chemotherapeutic molecules^[^ ^86^, ^87^, ^90^, ^91^ ^]^ or train the immune system to attack tumors.^[^ ^92^ ^]^ In this section we will discuss the limited number of approaches that have been developed to fight bacterial and viral infections. Despite the historical, current, and future global threat of viral pandemics, there is a strikingly large gap of work in this direction using rational nucleic acid‐based design. Therefore, we will also connect the dots between several studies published in the past decade, which point toward the promise of developing synthetic vaccine approaches against emergent viruses.

### 3.1 Delivery of Antisense Oligonucleotides to Bacteria

Often, the attachment of therapeutic agents to a nanoparticle‐based scaffold is a major synthesis challenge, requiring specialized conjugation chemistries or complicated purification steps to attain the final formulation. One class of therapeutics where using nanostructures constructed from DNA or other oligonucleotides offers a distinctive and straightforward solution is that of nucleotide‐based therapeutics. Including small interfering RNA (siRNA),^[^ ^93^ ^]^ miRNA,^[^ ^94^ ^]^ and antisense oligonucleotides (ASO),^[^ ^95^ ^]^ these therapeutic agents target gene regulatory or expression networks and typically comprise modified or chemically stabilized oligonucleotide variants that still obey typical Watson–Crick base pairing. Thus, their integration into DNA‐based nanostructures is a straightforward matter of exploiting the well‐known rules of generating complementary sequences. Here, we will discuss a handful of studies where nucleotide‐based therapeutics have been loaded onto simple, DNA‐based nanostructures to enhance their antibacterial activity. Despite their promise, we will not be covering self‐assembling nucleic acid conjugate materials, such as lipid–oligonucleotides,^[^ ^96^ ^]^ but rather focus on examples where a rational design approach was used to construct the underlying nanoparticle.

To tackle bacterial infections, ASOs have been the typical weapon of choice among studies involving gene regulation by DNA nanostructures. These can be used to specifically knock out some gene for antibiotic resistance or genetically inhibit some essential function related to pathogenicity. A fundamental prerequisite to using this strategy is the delivery of the ASO itself into the interior of the bacteria, meaning that any carrier must transport its payload through both its peptidoglycan cell wall and its lipid bilayer membrane. Hu and colleagues thoroughly examined the uptake of small tetrahedral DNA nanostructures into a collection of Gram‐positive (*S. aureus*) and Gram‐negative (*E. coli*, *Shigella flexneri*, *Klebsiella pneumoniae*, *Pseudomonas aeruginosa*, and *Acinetobacter baumannii*) bacteria.^[^ ^97^ ^]^ Even though fluorescently labeled tetrahedron carriers were shown to associate with all species of bacteria with higher instance than single‐stranded oligonucleotides, DNAse digestion followed by either flow cytometry or confocal laser scanning microscopy analysis indicated that only a small fraction successfully passed through into the interior. This could be improved by packaging the nanostructures in Lipofectamine 2000 (LP2000), a cationic, lipophilic transfection agent commonly used to shuttle nucleic acids into cells. However, this was only notable for Gram‐negative *E. coli*, which reached a peak entry efficiency of 83%, whereas only 40% entry could be reached for Gram‐positive *S. aureus*, likely due to the increased thickness of the peptidoglycan cell wall. The increase in entry efficiency into other bacteria—all Gram negative like *E. coli*—was not reported. The ability of the LP2000‐encapsulated nanostructures to transport phosphorothioate‐stabilized ASOs (PTO‐ASOs) into *E. coli* was further tested. In bacteria genetically transformed to express a green fluorescent protein (GFP), a 75% reduction in fluorescence was seen after treatment with nanostructures bearing an anti‐GFP ASO. More relevant to antimicrobial therapies, the nanostructure‐mediated transport of ASOs against the *acpP* gene critical for fatty acid synthesis into *E. coli* cells led to an ≈80% reduction in bacterial colonies when applied at 1 μM for 5 h.

**Figure 4:**
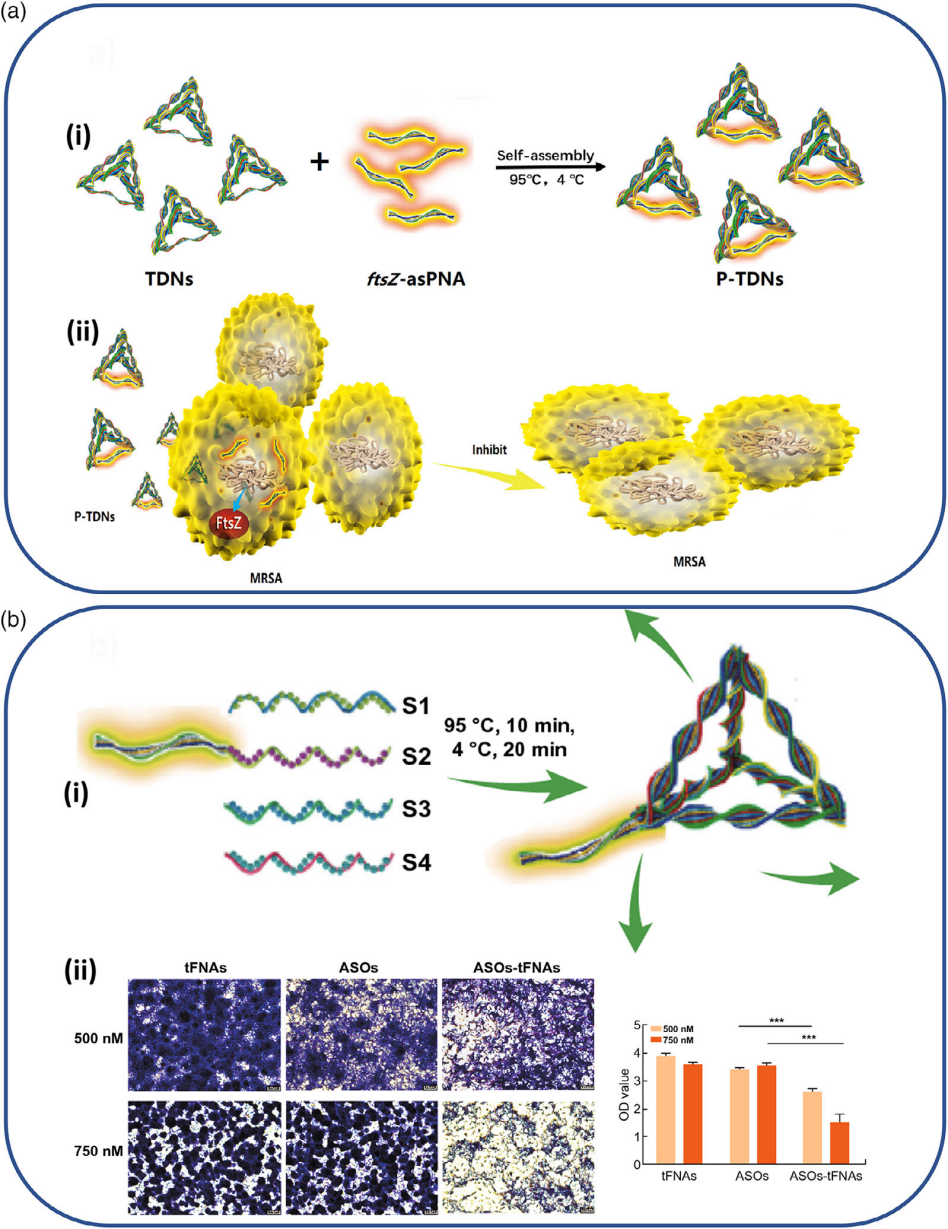
Strategies for the delivery of ASOs to bacteria. a) ASOs against the gene *ftsZ*, critical for replication of MRSA, (panel i) are synthesized from biostable PNA and hybridized on one edge of a DNA‐based tetrahedron nanostructure. (panel ii) Their application to MRSA cells in solution inhibits growth. Reproduced with permission.^[^ ^100^ ^]^ Copyright 2018, American Chemical Society. b) A PNA–ASO against three genes responsible for biofilm formation are (panel i) incorporated into a DNA tetrahedron, (panel ii) leading to a decrease of mature biofilm formation, as detected by crystal violet staining and optical density (OD) measurements. Reproduced with permission.^[^ ^102^ ^]^ Copyright 2020, Springer Nature.

Despite the aforementioned evidence that packaging into cationic lipophilic materials such as LP2000 significantly assists the delivery of ASO into target bacteria, several studies have nevertheless shown that “bare” DNA nanostructures are capable of delivering ASOs to disrupt their normal function or make them more susceptible to antibiotic treatments. A pair of studies integrated so‐called peptide nucleic acid or PNA—a DNA‐like material with the typical sugar phosphate backbone replaced with peptide bonds—as biostable ASOs into the structure of the otherwise dsDNA tetrahedron. When coassembled in this way, the DNA structure can potentially act as a bulky, protective scaffold around the therapeutic nucleic acid and also provide structural functionality for the addition of chemical moieties for enabling pathogen targeting or assisting in uptake. Upon digestion of the DNA part of the nanostructure by nucleases, PNA–ASO is freed to genetically inhibit a specific function within the bacteria. Using this strategy, Readman and colleagues overcame resistance in an *E. coli* strain (LREC461), that is known to be resistant to the widely used, broad‐spectrum antibiotic cefotaxime.^[^ ^98^ ^]^ PNA–ASO, named PNA4, which was previously shown to genetically counter this resistance in midmicromolar concentrations,^[^ ^99^ ^]^ was integrated into one of the edges of the tetrahedron by base pairing. In a concentration range between 10 and 30 μM, the PNA‐loaded tetrahedron was found to potentiate the inhibitory effect of 16 mg L^−1^ of cefotaxime in a dose‐dependent manner, compared with no effect with control tetrahedra with no PNA–ASO and similar concentrations of PNA–ASO in isolation. Furthermore, in the presence of 40 μM of PNA‐loaded tetrahedra, the minimal inhibitory concentration (MIC) of cefotaxime itself was generally lowered from 35 to 16 mg L^−1^. While the relatively high concentration of the DNA construct necessary to essentially overcome the natural resistance mechanism and ultimately induce antibiotic susceptibility is likely well beyond any range that would be feasible for pharmaceutical development, results reported by Zhang et al. showed that direct inhibition of a replication gene in resistant bacteria can significantly reduce the necessary concentrations.^[^ ^100^ ^]^ Again, a strategy was utilized where PNA–ASO, this time targeting the *ftsZ* gene involved in replication, was hybridized in a complementary fashion along one edge of the DNA tetrahedron nanostructure (see **Figure** **4**a). At a concentration of 750 nM, the tetrahedron structures were seen to enter methicillin‐resistant *S. aureus* (MRSA) with a moderate rate of 31.8% after 12 h. When applied to MRSA in increasing doses, a concentration‐dependent reduction of growth was seen after 24 h of treatment, with an inhibition rate of nearly 60% seen at a maximum concentration of 750 nM. Notably, inclusion of the PNA–ASO into the DNA nanostructure only led to moderately higher inhibition rates than its application in isolation. The roughly one‐third uptake efficiency into MRSA cells reported by the authors is likely a limiting factor for reaching lower effective doses, particularly in comparison with the PNA–ASO itself; however, the combination with either cationic transfection agents as described above^[^ ^97^ ^]^ or the integration of additional cell‐penetrating peptides might provide routes to improvement.

While specific structural or metabolic mechanisms to resist the effect of antibiotics is one major cause of concern, the formation of impenetrable bacterial biofilms on surfaces is also a collective mechanism that can lead to general resistance to a variety of typically bactericidal substances.^[^ ^101^ ^]^ Therefore, Zhang and colleagues also applied ASOs targeting three genes (*gtfBCD*, *gbpB*, and *ftf*) in *Streptococcus mutans* that are responsible for the secretion of polysaccharides necessary for their ability to form biofilms.^[^ ^102^ ^]^ Here, chemically stabilized ASOs consisting of either phosphorothioate‐modified DNA (PTO‐DNA) or 2′‐O‐Methyl RNA bases (2′‐O‐Me RNA) were extended from the vertex of the same tetrahedral DNA nanostructure already described above (see Figure 4b). Rather than directly inhibiting the growth of the bacteria, the constructs demonstrated a clear inhibition of mature biofilm formation, as directly imaged with crystal violet staining. After a 24  and 48 h treatment, samples containing the ASO that was transported on the tetrahedron at 750 nM had a nearly double effect in inhibiting biofilm formation compared with ASOs applied in isolation. Even though the results are very preliminary, this does provide another possible route to sensitize harmful bacteria biofilms, a major source of infection in medical facilities,^[^ ^103^ ^]^ to antibiotic treatments or other sanitization methods.

### 3.2 Delivery of Bactericidal Substances

Several strategies have also used DNA nanostructures as carriers for bactericidal substances that disrupt vital structures or functions of the targeted microbe, such as antibiotics, short peptide fragments, or enzymes. In many cases, the inherent affinity of DNA‐binding antibiotics or electrostatic attraction of cationic, membrane‐disrupting antimicrobial peptides (AMPs) to the nanostructure is leveraged to enable loading of the composite nanoparticle.

#### 3.2.1 Antibiotics

**Figure 5:**
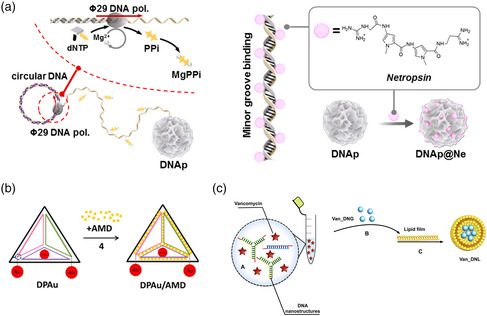
Strategies for the transport of antibiotics to bacteria by DNA nanostructures. a) The DNA‐binding antibiotic molecule netropsin is loaded into DNA “nanoflower” objects that are formed from a rolling‐circle PCR reaction. Reproduced under the terms of the Creative Commons Attribution 4.0 license.^[^ ^104^ ^]^ Copyright 2019, The Authors. b) The antibiotic actinomcycin D intercalates dsDNA and is loaded onto rigid DNA‐based tetrahedron structures. The additional attachment of gold NCs enables concurrent microscopic tracking of the nanostructures. Reproduced with permission.^[^ ^106^ ^]^ Copyright 2014, American Chemical Society. c) The positively charged glycopeptide antibiotic VAN is encapsulated within compact “DNA nanogels” formed from the polymerization of small y‐shaped and linear dsDNA monomers. The VAN‐loaded nanogels are surrounded by a protective lipid bilayer to ensure stability in physiological environments. Reproduced with permission.^[^ ^107^ ^]^ Copyright 2020, Elsevier B.V.

For antibiotic molecules such as vancomycin (VAN) or actinomycin, association with a DNA nanostructure offers a dual advantage through its ability to deliver a high local concentration to the point of action, together with a mechanism for the sustained release of the bactericidal substance through its natural degradation by nucleases. This type of slow release mechanism can already be attained by simple binding/unbinding kinetics, as shown in a recent study by Jeon et al., for the natural minor‐groove‐binding antibiotic netropsin.^[^ ^104^ ^]^ Here, large DNA “nanoflower” particles formed by condensed rolling‐circle PCR amplification products were successfully loaded with netropsin (see **Figure** **5**a), and its slow release into the solution was observed over the course of several days. While these micron‐sized particles were not applied to bacteria in the study, the recent observation that these same DNA nanoflower structures tend to co‐localize with bacterial and other types of pathogens following uptake by macrophages^[^ ^105^ ^]^ suggests a possible strategy for inhibiting infections such as tuberculosis that originate in the macrophage niche.

The enhancement of bactericidal effects by transporting antibiotics was reported in a pair of studies using either actinomycin D (AMD) or VAN, which were associated with two vastly different types of DNA nanostructures. Setyawati and coworkers used a strategy reminiscent of several of the already described studies, loading the DNA‐intercalating antibiotic AMD onto a rigid DNA tetrahedron and using its natural digestion by nucleases to release the drug within the cell interior (see Figure 5b).^[^ ^106^ ^]^ Each DNA nanostructure was determined to hold an average of 49 AMD molecules, representing a high local concentration, and were readily uptaken by both Gram‐negative *E. coli* and Gram‐positive *S. aureus* cells, albeit with a 20% higher efficiency for the latter. The AMD‐loaded nanostructures showed a higher concentration‐dependent inhibition of bacterial growth than an equivalent amount of free AMD in solution. The overall effect was more pronounced for *S. aureus*, with 100 μM of the nanostructures leading to a 65% inhibition in growth compared with 42% for an equivalent amount of AMD in solution. However, the enhancement of the effect was more evident in *E. coli*, where a mere 14% reduction by AMD alone was increased more than threefold to 48% inhibition when loaded onto the nanostructure. In a different strategy, Obuobi and coworkers used the cationic nature of the glycopeptide antibiotic VAN, considered to be a last‐resort treatment of severe indications such as sepsis, as the mechanism for loading it into lipid‐encapsulated DNA nanogels.^[^ ^107^ ^]^ The nanogels were formed by mixing branched, three‐arm Holliday‐junction DNA structures with short dsDNA linkers, which are interconnected with complementary sticky ends. By including an equal amount of VAN as monomeric DNA components and conducting a thermal annealing protocol, the drug could be electrostatically associated throughout the nanogels. They were subsequently encapsulated in a protective lipid bilayer comprising soy phosphatidylcholine (see Figure 5c). The resulting condensed structures were previously shown to form uniform nanoparticles with diameters ranging from roughly 30 to 250 nm, depending on the precise assembly conditions,^[^ ^108^ ^]^ and swelled to approximately double that size when loaded with VAN. This formulation showed a sustained release under physiological conditions, with approximately one‐third and one‐half of the VAN released into the surrounding after 12 and 24 h, respectively. This was increased to 85% after 24 h when exposed to a lipase enzyme, such as those secreted by the target pathogen *S. aureus*. A concentration‐dependent inhibition of *S. aureus* growth was seen in the range of several μg ml^−1^; however, the results were similar to both free VAN in solution and a simpler formulation of the nanogel that was not encapsulated in a lipid vesicle. Nevertheless, a stronger comparative antimicrobial effect was seen when the lipid‐encapsulated nanogel formulation was used as a treatment on macrophages that were infected with *S. aureus*. While a 1 μg ml^−1^ concentration led to a 2.35 log reduction of colony‐forming units (CFU) after 24 h, a significantly inferior 0.21 log reduction was seen for an equivalent amount of free VAN. Already at concentrations of 10 and 100 μg ml^−1^, the lipid‐encapsulated nanogels were able to inhibit more than 99% of intracellular bacteria growth.

#### 3.2.2 Antimicrobial Peptides and Enzymes

AMPs against bacteria typically target their protective lipid membrane and act by either generally destabilizing it or forming pore‐like transmembrane channels.^[^ ^109^ ^]^ As many of these are highly cationic in nature, they electrostatically bind to the dense negative charges of dsDNA. Therefore, they are attractive options for straightforward loading onto or packaging within different types of DNA nanostructures. Previous methods have utilized DNA aptamers that specifically bind to certain polypeptide motifs appended to the end of AMPs as biodegradable linkers to inorganic nanoparticles.^[^ ^51^ ^]^ However we will focus here on cases where a DNA‐based architecture is used as the means of transport or encapsulation.

**Figure 6:**
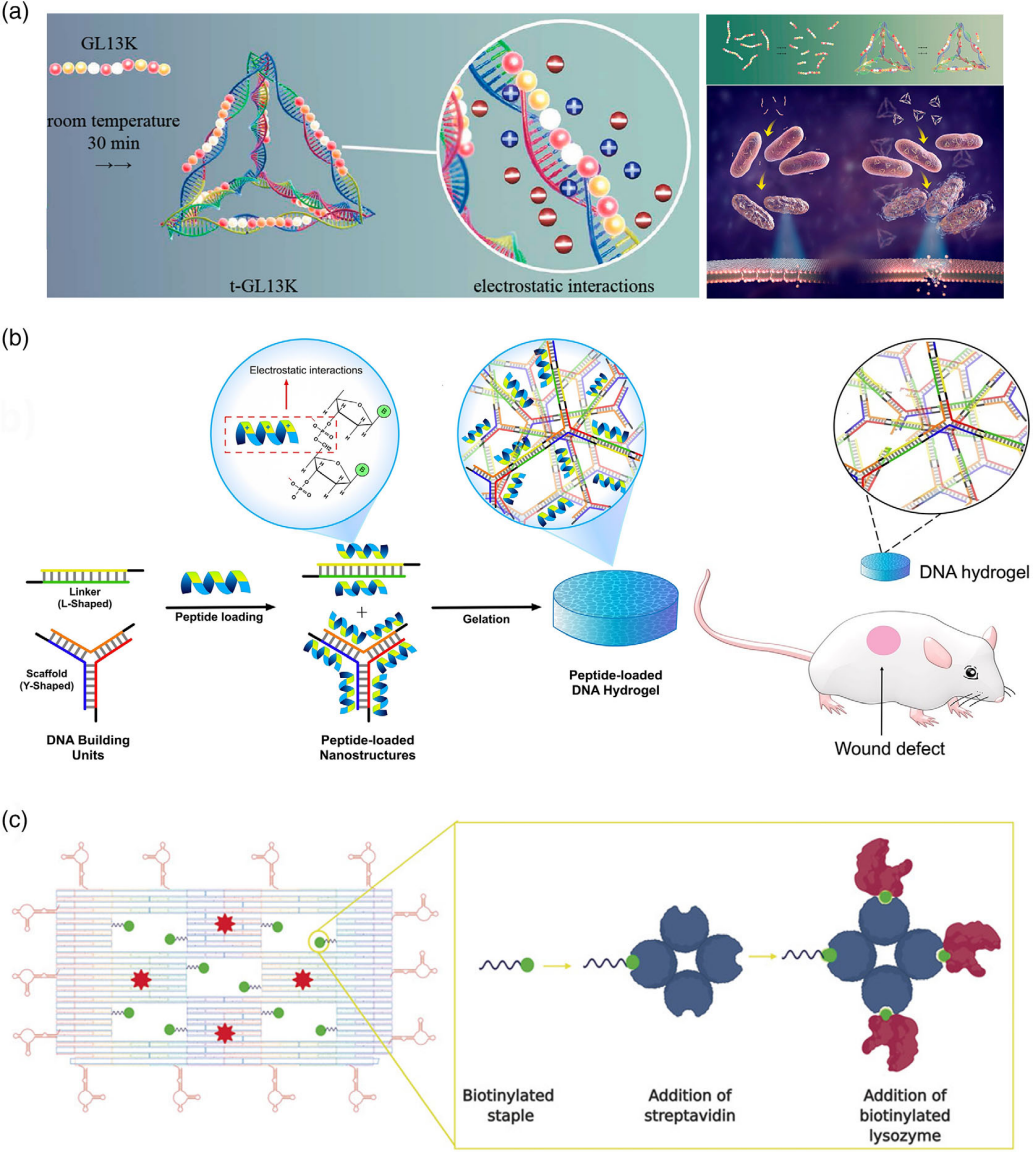
Transport of peptide‐ and enzyme‐based bactericidal substances. a) An AMP known to disrupt the membrane of several species of bacteria is electrostatically attached to the edges of a DNA tetrahedron. This leads to an enhanced antibacterial rate in species known to be susceptible as well as those that are previously shown to be resistant to the AMP. Reproduced with permission.^[^ ^110^ ^]^ Copyright 2020, American Chemical Society. b) A DNA‐based hydrogel is loaded with a broad‐spectrum AMP and used as a wound dressing in in vivo experiments, reducing healing times. Reproduced with permission.^[^ ^112^ ^]^ Copyright 2019, Elsevier B.V. c) A bactericidal enzyme, lysozyme, is loaded in high amounts onto a DNA origami nanostructure, together with aptamers that target specific Gram‐negative and Gram‐positive bacteria. Reproduced with permission.^[^ ^115^ ^]^ Copyright 2020, John Wiley and Sons.

The frequently‐used DNA tetrahedron nanostructure was the underlying structure in a study by Liu et al., where association with an AMP seemed to play a protective role in delivering the payload.^[^ ^110^ ^]^ Cationic AMP GL13K, which is known to disrupt the negatively charged bacterial cell membrane,^[^ ^111^ ^]^ was combined at high ratios with the 10 nm DNA tetrahedra, together forming DNA–peptide polyplexes up to 150 nm average diameter (see **Figure** **6**a). Moderate ratios that seemingly led to the formation of individual or oligomeric units consisting of a small number of DNA constructs were then applied to either *E. coli* or *Porphyromonas gingivalis*. While the former is known to be susceptible to GL13K, the latter resists disruption to its cell membrane due to the local secretion of proteases. As expected, *E. coli* were already highly susceptible to free GL13K with an antibacterial rate of 85%; however, this was increased to levels indicative of complete inhibition (antibacterial rate 99%), when the combined DNA–peptide polyplexes were applied. Remarkably, the structures also led to the emergence of moderate antibacterial activity in the usually resistant *P. gingivalis* samples, where an increase of the antibacterial rate from 1% to 32% was observed. Mass spectrometry analysis of recovered AMPs show that this is likely due to a decreased degradation when they were complexed together with the 3D DNA nanostructure.

Two studies from Obuobi et al. utilized the same DNA‐based nanogel formulation described above for VAN delivery, however, substituted the positively charged glycopeptide antibiotic with a broad‐spectrum AMP, L12.^[^ ^112^, ^113^ ^]^ This AMP was previously shown to be effective against a large number of bacteria, including both drug‐susceptible and multidrug‐resistant species.^[^ ^114^ ^]^ A similar assembly approach was used as described earlier; however, subtle alterations led to either a condensed DNA hydrogel suitable for application as a wound dressing^[^ ^112^ ^]^ or discrete DNA nanogels that could be locally applied to eye infections.^[^ ^113^ ^]^ The hydrogel formulation was able to effectively kill MRSA, *E. coli* and *P. aeruginosa* in in vitro tests, and were particularly effective against MRSA, likely due to the species’ natural secretion of high amounts of nucleases leading to quicker release of the AMP (see Figure 6b). The antibacterial properties of the hydrogel as a potential wound dressing were tested in an ex vivo explant model, consisting of sections of porcine skin that was infected with *S. aureus*. They were shown to have a 4 log reduction in bacteria 24 h after treatment compared with hydrogels without AMPs. Finally, when applied to postoperative mouse wounds, the AMP‐loaded hydrogels led to a faster healing time and marked reduction in typical signs of posthealing tissue damage. Showing the versatility of this method, discrete, compact nanogels of 100–500 nm were also loaded with AMP‐L12 and used as a treatment of bacterial keratitis or simply, bacterial‐induced inflammation of the eye, in mice.^[^ ^113^ ^]^ Again, the same panel of three bacteria was used to confirm the formulations ability to release the AMP into solution and effectively inhibit their growth, and additional in vivo validation was conducted with *S. aureus*. An artificial wound was generated in the eyes of mice by removing part of the cornea, they were treated with the bacteria, and the DNA nanogel formulations were applied 6 h postinfection. Results were compared with usual clinically approved treatment of 0.3% w/v gatifloxacin. While corneal opacity, a sign of the progression of the infection, was less significantly reduced after treatment with DNA nanogel formulations than gatifloxacin, a significantly greater reduction of inflammation was seen after 24 and 48 h, likely a result of earlier‐discovered anti‐inflammatory properties of these structures.^[^ ^112^ ^]^ Overall, the reduction of bacterial burden after 48 h was comparable for 0.3% gatifloxacin and an equivalent amount of AMP‐loaded DNA nanogels, indicating the potential for this strategy to be further optimized and developed as a candidate therapy.

A far more complex approach was reported by Mela et al., who constructed a truly multimodal DNA origami system capable of targeted delivery of a bactericidal enzyme.^[^ ^115^ ^]^ Here, lysozyme was used as a known agent capable of breaking down the peptidoglycan Gram‐positive cell wall. While it is less effective against Gram‐negative bacteria due to their outer lipid membrane, it nevertheless shows some efficacy when it is specifically targeted to the bacterial surface. To achieve broad‐spectrum efficacy, up to ten streptavidin proteins were used as a multivalent anchor points on biotinylated staple strands integrated into “windows” of the DNA origami structure, and as many as three biotinylated lysozymes were attached to each of these anchor points (see Figure 6c). The edges of the sheet‐like DNA origami surface were decorated with 14 aptamers known to target *E. coli* and *B. subtilis* strains,^[^ ^116^ ^]^ and a dye molecule was also included for detection. The compound DNA origami construct did lead to a significantly amplified inhibitory effect when applied to the Gram‐positive *B. subtilis*, with an aggregate amount of 300 nM of lysozyme on the structures causing a significant reduction in growth over 16 h compared with relatively little effect from an equivalent amount of free enzyme. In contrast, while the empty DNA origami structure curiously showed a significant reduction of bacterial growth for *E. coli*, likely due to some physical interference with bacterial growth and/or division, the addition of enzyme to those structures had no effect, confirming its natural resistance to degradation.

### 3.3 Inhibition of Viral Infections

Despite the historical, current, and certain future threats of localized outbreaks and global pandemics from viruses such as influenza, Zika, coronavirus, and others, surprisingly few studies have focused on therapeutic or inhibitory approaches using rationally designed DNA nanostructures. Often, the high biosafety requirements for working with transmissible viral pathogens limits researchers’ ability to pursue work with viable viral particles due to the need for specialized infrastructure and trained personnel. Nevertheless, recent work using, for example, inorganic structural scaffolds to arrange and amplify the effect of virus inhibitors^[^ ^117^ ^]^ points toward the relevance for a DNA‐ or nucleotide‐based approach.

**Figure 7:**
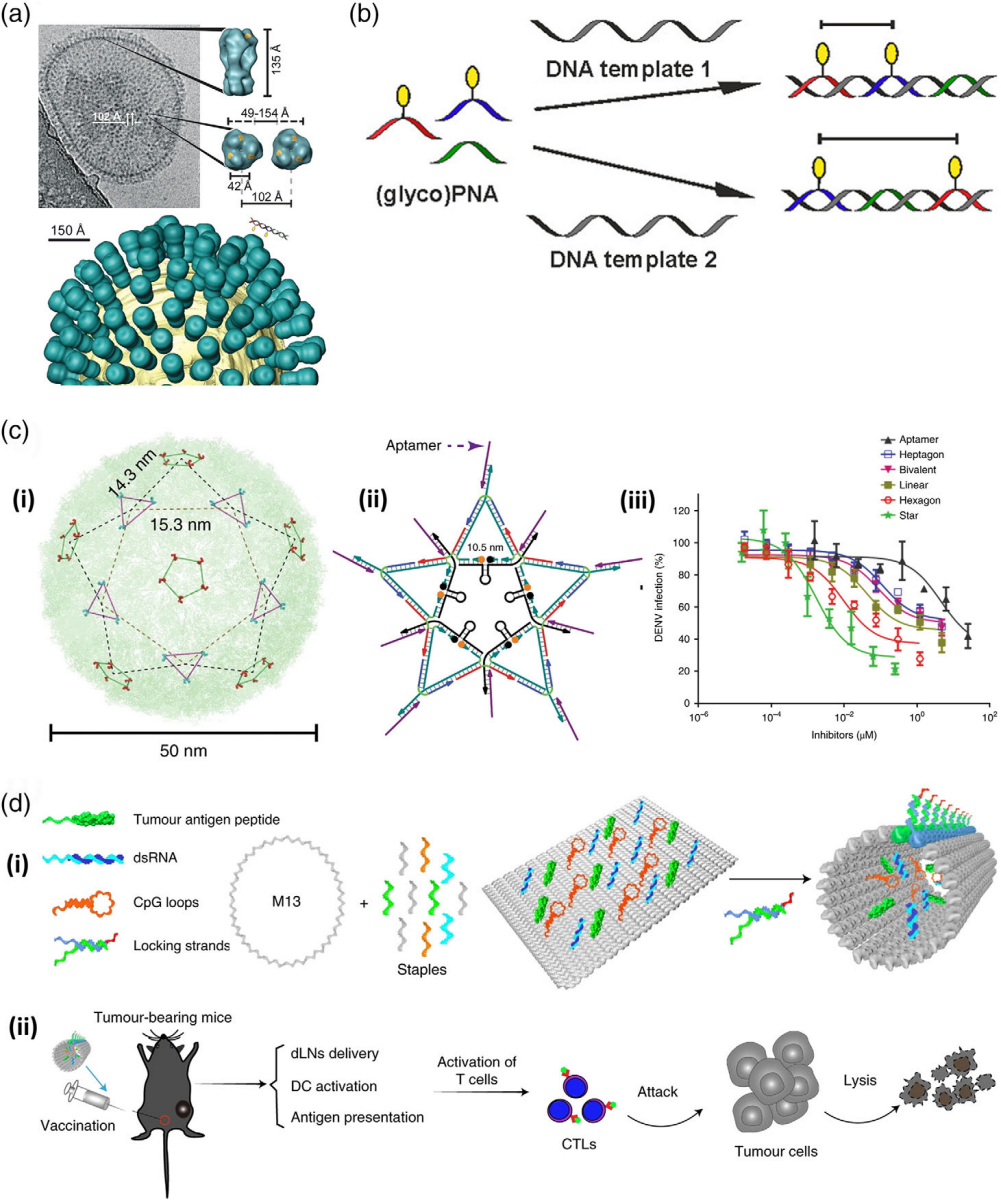
Current and possible application of DNA‐based nanostructures to fight viral infections. a) The outer surface of the IAV is covered with a large number of HA receptor proteins, which are responsible for binding to host cells. These are homotrimers, comprising three identical subunits. b) Oligonucleotide‐based templates, here a DNA–PNA hybrid, can be used to enable spatially dependent bi‐ or multivalent binding of small ligands (here, a sialic acid derivative known to bind to influenza HA) to targeted virus proteins. (a,b) Reproduced with permission.^[^ ^121^ ^]^ Copyright 2017, American Chemical Society. c) The outer surface of the DENV is covered with (panel i) regularly spaced clusters of ED3 receptor proteins. (panel ii) By designing a geometrically complementary DNA nanostructure that bears aptamers that block DENV's ability to infect cells, (panel iii) a high antiviral activity can be achieved, compared with other designs with less similarity to the ED3 cluster distribution. Reproduced with permission.^[^ ^124^ ^]^ Copyright 2019, Springer Nature. d) A DNA origami‐based nanovaccine (panel i) is based on the patterning of adjuvants (dsRNA and CpG loops) and tumor antigen peptides onto a stimulus–responsive nanostructure. (panel ii) When injected into living mice, the immune system is stimulated to produce CTLs that attack later challenges with tumor cells. Reproduced with permission.^[^ ^92^ ^]^ Copyright 2020, Springer Nature.

The few instances of this type of approach being used against viruses have focused on physically blocking the proteins on the virus surface that are responsible for binding to structures on the surface of host cells and enabling the transfer of their genetic material into the interior. This so‐called antiadhesive or fusion‐blocking strategy roughly mimics the effect of naturally occurring neutralizing antibodies produced by the immune system.^[^ ^118^ ^]^ As these proteins typically comprise two, three, or more individual subunits and contain multiple binding domains arranged in a precise geometrical orientation relative to each other (e.g., the homotrimeric hemagglutinin [HA] protein of IAV)^[^ ^119^ ^]^ (see **Figure** **7**a), strategies using the multivalent presentation of ligands can be used to rationally design inhibitors.^[^ ^120^ ^]^ A study by Bandlow et al. already hinted at the potential of using the nucleic acid‐based arrangement of ligands to enhance antiviral efficacy (see Figure 7b).^[^ ^121^ ^]^ Pairs of a trisaccharide sialic acid derivative, the natural ligand for IAV HA, were presented on rigid, dsDNA–PNA segments at different interligand spacings ranging from 23 to 101 Å. A significant enhancement of binding activity was observed for interligand distances close to the 42 Å distance between binding pockets previously reported from crystal structure analysis. When compared with a single, monovalent ligand on a DNA–PNA segment, bivalent presentation in the range of 42–59 Å showed enhancement factors of at least 30–50*x* in the binding constants to native and cleaved HA and the ability of the construct to inhibit the intact virus from agglutinating red blood cells. Using RCA to generate an arbitrarily large single‐stranded DNA template with complementary binding sites for bivalent arrangements of the trisaccharide sialic acid derivative with optimal 42–59 Å spacing, they later showed in a follow‐up study that massively multivalent particles could inhibit red blood cell agglutination in nanomolar concentrations.^[^ ^122^ ^]^ A trio of studies by Yamabe and colleagues examined the impact of trimeric sialic acid arrangements on binding to different IAV strains through the use of simple three‐way Holliday junctions or triangular DNA nanostructures, each formed by the assembly of three oligonucleotides.^[^ ^123^ ^]^ These structures were designed to roughly match the geometrical spacing between the three HA binding pockets of the trimeric protein, with each of the three presenting arms displaying between one and five sialic acid molecules. In all cases, inhibition of red blood cell agglutination was observed to occur in the mid‐to‐high nanomolar range, an improvement of roughly five orders of magnitude over monomeric sialic acid.

It should be noted that the studies described above only demonstrated the ability of functionalized DNA nanostructures to inhibit ligand binding by the virus, which does not necessarily correlate with their ability to inhibit the virus from infecting host cells. In each case, the analysis was based on the binding of virus particles to the surfaces of red blood cells, without any subsequent internalization, whereas epithelial cells are the typical targets for infection by influenza. While this sort of binding assay is a good way to gauge how a particular inhibitory construct can generally interfere with the physical interaction between a virus and its natural ligand, a myriad of other factors ultimately determines whether this translates to actual inhibition of infection. Below, we thus will focus on the few instances where inhibition of infection was successfully achieved.

This basic principle of geometrically arranging inhibitory ligands on DNA nanostructures was utilized by Kwon and colleagues to inhibit the mosquito‐transmitted flavivirus dengue virus (DENV).^[^ ^124^ ^]^ Their approach involved the placement of up to 10 DENV‐blocking aptamers on a 2D, star‐shaped construct, assembled from 21 synthetic oligonucleotides using a tile‐based construction method (see Figure 7c). Here, the regular matrix‐like arrangement of clusters of the surface protein responsible for attachment of DENV to the host cell (envelope protein ectodomain III, ED3) was used to design the star‐shaped structure, with each of the ten aptamer‐displaying vertices corresponding to the location of a tri‐ or pentavalent ED3 cluster. These aptamers are known to selectively bind and inhibit the ED3 of all four DENV serotypes. With the help of a fluorophore/quencher scheme, the DNA‐based construct was able to sense DENV in human serum and plasma with a LOD of 100 and 1000 plaque‐forming units per milliliter (PFU ml^−1^), respectively. This proved to be equivalent or better than the gold standard PCR‐based detection method and well within the range necessary to detect typical viral loads in patients at the onset of symptoms. More importantly, the aptamer‐loaded DNA nanostructures were also found to inhibit the ability of DENV to enter and infect host cells with a similarly dramatic effect. Whereas the aptamer itself was previously reported to inhibit infection with a half maximal inhibitory concentration (IC_50_) of ≈15 μM, presentation on the star‐like construct led to a 7500‐fold reduction to only 2 nM, as shown by standard in vitro plaque reduction assays. This reduction was indeed due to the geometrically complementary arrangement of aptamer ligands on the star‐shaped structure, as its presentation on hexagonal, heptagonal, and flexible linear DNA nanostructure scaffolds led to moderately inferior IC_50_ values of 10, 440, and 90 nM, respectively.

While these structures were all stable in human blood serum and plasma, any in vivo therapeutic applications would certainly require the implementation of stabilized DNA derivatives or DNA‐like Xeno‐nucleic acids^[^ ^125^ ^]^ that are resistant to nucleases or other mechanisms of the mammalian immune system. Encouragingly, the rigid bivalent presentation of only two aptamers was also surprisingly effective when taken in comparison with the monovalent aptamer, with an IC_50_ value of 130 nM still providing more than a 100‐fold improvement in inhibition. Due to the comparative simplicity and lower cost of mass‐produced short ds segments of modified oligonucleotides compared with large, complex structures comprising multiple branches and hundreds of bases, this could point toward a strategy suitable for industrial‐scale pharmaceutical development.^[^ ^34^ ^]^ To this end, two recent patent filings, in 2017 and 2019, cover a nucleic acid‐based approach for enhancing the efficacy of virus‐inhibiting peptides.^[^ ^126^, ^127^ ^]^ The supporting data reported relatively simple DNA nanostructures typically composed of three oligonucleotides, containing a single branch point, displaying the inhibitory peptides in a geometrical complement to the binding pockets on a single virus surface protein. The strategy was shown to generally improve binding strength and/or virus inhibition in IAV, DENV, and respiratory syncytial virus (RSV), by enhancing the activity of peptides targeted to the binding pockets of their HA, ED3, and fusion protein (RSV‐F), respectively. The most promising effect was reported for nanostructures targeting RSV‐F, where the trivalent presentation of the peptide enhanced the inhibitory effect by several hundred‐fold.^[^ ^126^ ^]^

Despite the apparent dearth of studies on direct inhibition of viral infections enabled by nanostructures constructed from DNA or other nucleotide‐based materials, we can still speculate where prior work in the field gives hints of where new approaches could arise. With the recent attention to the rapid development of vaccines to alleviate the COVID‐19 pandemic we will highlight a selection of earlier work on DNA‐based approaches that, taken together, provide a roadmap to the development of future antiviral vaccines. Historically, most clinically approved vaccines or those undergoing clinical development are based on attenuated or inactivated viruses^[^ ^128^ ^]^ or antigenic fragments derived from viral proteins or their surface glycosylation.^[^ ^129^ ^]^ Often, additives known as adjuvants are concurrently applied to stimulate or prime the immune system to be more active in developing a programmed response to the viral antigen. More recently, nanoparticle‐based approaches have come into focus as the means for exploiting the useful concepts of rational design and modular construction to maximize their effect in training the immune system against a certain target,^[^ ^130^ ^]^ potentially enabling the spatial linking or complex geometrical arrangement of antigen and adjuvant to cause a stronger synergistic response.

Using nucleic acid‐based nanostructures as the means to present antigenic proteins or adjuvants to immune cells is by no means a recent concept. Studies dating back to 2008 utilized simple Y‐shaped,^[^ ^131^ ^]^ dendritic,^[^ ^132^ ^]^ or polypod^[^ ^133^ ^]^ wireframe DNA‐based nanostructures as carriers of multiple so‐called CpG motifs or unmethylated cytosine–phosphate–guanine dinucleotides known to stimulate immune responses through activation of the TLR9 receptor pathway.^[^ ^134^ ^]^ This was extended to more complex 3D DNA nanostructures in a pair of 2011 studies, by incorporating the motifs either onto a rigid wireframe tetrahedron^[^ ^135^ ^]^ or a barrel‐shaped DNA origami.^[^ ^136^ ^]^ While all cases demonstrated that the collective effect of linking multiple adjuvant molecules together on a single scaffold amplifies their effect, or simply, that the whole is greater than the sum of its parts, the results of the latter two were particularly instrumental in subsequent studies in vaccine development.

The oft‐utilized DNA tetrahedron carriers used above by Li and coworkers^[^ ^135^ ^]^ were later used in a study by Liu et al., where four adjuvant CpG sequences placed at each of the vertices were combined with a model antigen—the protein streptavidin—which was encapsulated in the interior.^[^ ^137^ ^]^ This synthetic vaccine nanoparticle was able to successfully induce a strong antibody response in mice against streptavidin following a two‐dose immunization protocol, which significantly exceeded that following similar immunization with unlinked CpG motifs and streptavidin. While limited to a nonclinically relevant target, this nevertheless demonstrated the potential for the modular construction of the necessary components on a DNA scaffold. In contrast, the study by Schüller et al. utilizing the barrel‐like DNA origami structure^[^ ^136^ ^]^ examined the impact of creating local high densities of adjuvants to significantly amplify immune activation, in their case with up to 62 CpG motifs placed on the 80 nm object. Indeed, the structures caused a strong immunostimulatory ex vivo response in primary mouse spleen cells, far greater than that of an equivalent of nonlinked motifs. A later connected study by Sellner and colleagues^[^ ^138^ ^]^ demonstrated the strength of this approach in living organisms, where the injection of DNA‐tile‐based nanotubes displaying 20 CpG motifs into the cremaster muscle of mice led to an almost immediate internalization of the nanostructures into macrophages, the subsequent recruitment of protective white blood cells to the tissue, and activation of the NF‐kB pathway in nearby cells. All three of these factors are important initially in the adaptive immune system's response to foreign threats and the development of immunity. As such, these are important prerequisites when evaluating the potential of an approach for vaccine development.

These two strategies of modular antigen adjuvant proximity coupling and high‐density adjuvant clustering were finally combined in a study by Liu et al., where a multifunctional DNA nanostructure was generated as a synthetic, antitumor vaccine (see Figure 7d).^[^ ^92^ ^]^ Similar to the earlier study by Schüller et al., a barrel‐shaped DNA nanostructure itself was assembled via the DNA origami method; however, this case included several critical differences. Here, stimulatory adjuvant molecules (both CpG motifs and dsRNA segments to activate both TLR3 and TLR9) as well as the tumor antigens (synthetically produced peptide segments) were all arranged in the inner surface of the barrel structure, which was held closed by a pH‐sensitive dsDNA‐locking system. This allowed the entry and stimulus–responsive release of antigens and adjuvants inside antigen‐presenting cells located in the lymph nodes of mice, which were sufficient to stimulate the production of tumor‐specific cytotoxic T lymphocytes (CTLs). This led to not only the short‐term prevention of tumor growth and metastasis in inoculated mice who were challenged with a melanoma model (B16‐OVA) but more interestingly long‐term immunity against antigen‐bearing melanoma cells that were injected 110 days following vaccination.

While the above examples are not in any way focused on programming immunity against viral infections, we suggest that this body of work, particularly in light of the successful in vivo demonstration of an antitumor vaccine, provides a clear roadmap for enabling future studies in synthetic vaccines against not only viruses but also pathogenic microorganisms in general. The modularity of these systems is of key importance, as small, synthetic antigens for a variety of different viruses or pathogens can be easily interchanged at will. Conjugation techniques for attaching peptides to different types of DNA nanostructures are now well‐established^[^ ^88^, ^139^ ^]^ and can be directly transferred to synthetically produced polysaccharide antigens or glycopeptide chimera of the two. As high‐quality structural studies combined with in silico modeling can even provide candidate antigenic sequences of new threats within a few months of their emergence,^[^ ^140^ ^]^ the establishment of pipelines effective for rational design and molecular construction of vaccines against viral threats could eventually become part of a rapid response against new threats.

## 4 Conclusions and Outlook

Biomedical DNA nanotechnology has made tremendous progress in the past decade and numerous diagnostic assays and therapeutic strategies utilizing a multitude of different DNA nanostructures and modification schemes have been reported in literature. While the early years have seen an almost exclusive focus on cancer treatment and diagnosis, we now seem to have reached a branch point. Even though the majority of recently published papers in the field is still dealing with cancer, more studies are exploring applications of biomedical DNA nanostructures in the treatment and diagnosis of other diseases such as acute kidney injury^[^ ^141^ ^]^ or Alzheimer's disease.^[^ ^142^ ^]^ As we have tried to convey in this review, the field of infectious diseases represents a particularly exciting and promising application area of biomedical DNA nanotechnology, even though we still stand at the very beginning of its exploration and can barely anticipate all the challenges and hurdles that lay ahead and all that may be gained by overcoming them.

In the area of diagnostics, most if not all DNA nanostructure‐based concepts for biomarker detection can also be adapted in one way or another to pathogen targets. Naturally, pathogen‐specific nucleic acids represent the first choice of target molecules, as they can easily be captured by complementary probe sequences protruding from the DNA nanostructure surface. In contrast, detection of protein biomarkers and whole pathogens requires the incorporation of suitable capture probes such as aptamers or antibodies, which may or may not be available for the target pathogen in question. While many of such DNA nanostructure‐based detection concepts have demonstrated high target sensitivity and selectivity under lab conditions, it is often not clear whether they are compatible with point‐of‐care diagnostic schemes or rather require complex sample processing, purification and target amplification steps. In contrast to cancer diagnostics, for instance, which is typically performed in well‐equipped bioanalytical labs and hospitals, rapid point‐of‐care testing plays a highly important role in controlling infectious disease outbreaks, in particular in remote areas.^[^ ^143^ ^]^ Nevertheless, a few of the pathogen‐sensing concepts discussed in this review have already been shown to require only minimal preprocessing of clinical samples,^[^ ^74^, ^81^ ^]^ which renders these concepts particularly promising for future applications in point‐of‐care diagnostics.

When it comes to therapeutic approaches, the situation is less clear. Whereas several studies have explored the potential of DNA nanostructure‐based packaging and delivery of ASO and various bactericidal substances to enhance their antibacterial efficacy, the results were often rather mixed. Even though the general concept of using DNA nanostructures to transport antimicrobial substances to or beyond the bacterial cell envelope has been shown to work in several preliminary investigations, the overall improvements to antimicrobial efficacy are often only moderate and accompanied by strong and hard‐to‐rationalize species dependencies. Taking into account the increased complexity, regulatory burden, and cost associated with producing and implementing even the simplest of synthetic, DNA‐based scaffolds on the industrial scale, their impact on the therapeutic efficacy will likely need to be nothing short of a paradigm shift when compared with current standards. Therefore, while this concept appears certainly promising, further systematic investigations will be needed to assess its full clinical potential. Furthermore, the majority of studies on the DNA nanostructure‐based delivery of bactericidal substances so far have focused on cationic molecules that can easily be complexed with DNA via nonspecific electrostatic interactions or specific binding to DNA (e.g. intercalation). While this is rather straightforward and does not require additional modifications of the molecules or the DNA nanostructures, the positive charge of the molecules is often a key factor in their interaction with the highly negatively charged bacterial cell membranes. Complexing these molecules with negatively charged DNA nanostructures may thus result in reduced efficacy and/or specificity. Alternative loading strategies and in particular the concept of multivalent drug presentation via covalent coupling should thus be explored in the future. Furthermore, antimicrobials whose effects are based on direct interactions with DNA do still retain their potential off‐target cytotoxicity; however, in this case the association with DNA‐based scaffolds does offer a direct advantage as additional targeting moieties can be integrated in a straightforward manner.

Perhaps the greatest opportunity for developing impactful, new approaches is a result of the relative lack of work done so far on using DNA nanostructures as the means to tackle infections of viral origins. Direct inhibition of viruses themselves is a subtly complex problem as they have no metabolic processes on their own but rely on hijacking that of their host cells after mounting a successful invasion. This means that any virucidal drugs whose mechanism of action depends upon disrupting specific points in their replication cycle by necessity also act upon off‐target cells of the host organism. The current effort of developing antiviral, regulatory RNA molecules such as siRNA to genetically inhibit infections^[^ ^144^ ^]^ does present a natural, if not obvious, opportunity to repurpose existing approaches for targeting DNA nanostructures to human cells. However, the inherently regular, geometrically defined, nanometer‐scale structure of the viral surface—both in terms of binding pockets on multimeric surface proteins and in terms of clusters thereof—has inspired approaches that exploit the most unique advantage of nucleotide‐based programming. Even though these are only at the proof‐of‐concept stage, using DNA nanostructures as the basis for patterning geometrically complementary arrangements of antiadhesive agents to physically block the binding and fusion of viruses to their host cells has shown promise for blood‐borne viruses like DENV^[^ ^124^ ^]^ and respiratory viruses such as RSV or influenza.^[^ ^126^, ^127^ ^]^ It is questionable as to whether these approaches would alleviate the complications from ongoing viral infections; however, their value could be achieved through use as prophylactics administered to potentially high‐risk individuals or front‐line care workers. In contrast, the use of DNA‐based fabrication approaches for designing synthetic vaccines against viruses is still unreported in published literature; however, a progression of previous studies over the past decade has drawn a clear roadmap for implementation. The recently reported construction of an antitumor vaccine based on the nanotemplating of multiple elements onto DNA origami is a first proof of concept, albeit for a vastly different class of target.^[^ ^92^ ^]^ Nevertheless, the full modularity of this approach, where different antigens and immune modulators can be linked together in nearly any combination, is particularly enticing for viruses, which can mutate from season to season and present new strains or serotypes with little warning or time to react.

Only one study^[^ ^80^ ^]^ discussed in this review focused on parasitic pathogens, i.e., the detection of a protein expressed by *Plasmodium falciparum*, the most severe cause of malaria.^[^ ^145^ ^]^ The DNA nanostructure‐based detection of parasite‐specific biomarkers in clinical samples thus appears feasible. Possible applications of DNA nanostructures in antiparasitic drug delivery, however, are harder to predict because of the large diversity and heterogeneity in this class of pathogens. Even though many studies demonstrated successful nanoparticle‐based inhibition of diverse parasites including *Plasmodium*, *Leishmania*, and tapeworm, the challenges are manifold.^[^ ^146^ ^]^ Parasites for instance have more complex life cycles than bacteria, with individual stages of the life cycle usually presenting different susceptibilities to a given drug. Furthermore, many parasites such as *Plasmodium* and *Leishmania* reside inside host cells and are therefore difficult to target. Whether DNA nanotechnology can stand up to these challenges remains to be seen.

While the spread and severity of invasive fungal infections have not been appreciated for a long time,^[^ ^13^ ^]^ the recent worldwide appearance of multidrug‐resistant fungal pathogens such as *Candida auris* ^[^ ^147^ ^]^ has resulted in increasing awareness.^[^ ^12^ ^]^ Consequently, nanoparticle‐based delivery of antifungal agents is receiving more attention from the nanomedicine community.^[^ ^148^ ^]^ However, we are not aware of any study so far that utilized DNA nanostructures in an attempt to detect or inhibit fungal pathogens. Nevertheless, we speculate that the great versatility of DNA nanotechnology may also aid in the fight against drug‐resistant fungal infections, for instance, in the detection of *C. auris* in patient samples, which is frequently misidentified in routine microbiological testing.^[^ ^147^ ^]^

In summary, we firmly believe that DNA nanotechnology has the potential to produce formidable and powerful weapons for the fight against infectious diseases. Many of the basic aspects of DNA nanostructures that have been uncovered in the past decade, for instance regarding stability,^[^ ^149^ ^]^ cellular uptake,^[^ ^150^ ^]^ and immune response,^[^ ^136^ ^]^ but also with respect to mass production^[^ ^151^ ^]^ and storage,^[^ ^152^ ^]^ will without doubt be of tremendous value also in this endeavor. Due to the great variety of infectious diseases and the high diversity of viral, bacterial, fungal, and parasitic pathogens, however, the road ahead will also present many challenges that have to be overcome to establish biomedical DNA nanotechnology also in the area of infectious disease diagnostics and therapy.

## Conflict of Interest

The authors declare no conflict of interest.
